# OsPEX11, a Peroxisomal Biogenesis Factor 11, Contributes to Salt Stress Tolerance in *Oryza sativa*

**DOI:** 10.3389/fpls.2016.01357

**Published:** 2016-09-15

**Authors:** Peng Cui, Hongbo Liu, Faisal Islam, Lan Li, Muhammad A. Farooq, Songlin Ruan, Weijun Zhou

**Affiliations:** ^1^Institute of Crop Science and Zhejiang Key Laboratory of Crop Germplasm, Zhejiang UniversityHangzhou, China; ^2^College of Agriculture and Food Science, Zhejiang A & F UniversityLin’an, China; ^3^Laboratory of Plant Molecular Biology and Proteomics, Institute of Biotechnology, Hangzhou Academy of Agricultural SciencesHangzhou, China

**Keywords:** *Oryza sativa*, protein interaction, *OsPEX11*, transgene, salt tolerance

## Abstract

Peroxisomes are single membrane-bound organelles, whose basic enzymatic constituents are catalase and H_2_O_2_-producing flavin oxidases. Previous reports showed that peroxisome is involved in numerous processes including primary and secondary metabolism, plant development and abiotic stress responses. However, knowledge on the function of different peroxisome genes from rice and its regulatory roles in salt and other abiotic stresses is limited. Here, a novel prey protein, OsPEX11 (Os03g0302000), was screened and identified by yeast two-hybrid and GST pull-down assays. Phenotypic analysis of *OsPEX11* overexpression seedlings demonstrated that they had better tolerance to salt stress than wild type (WT) and OsPEX11-RNAi seedlings. Compared with WT and OsPEX11-RNAi seedlings, overexpression of *OsPEX11* had lower level of lipid peroxidation, Na^+^/K^+^ ratio, higher activities of antioxidant enzymes (SOD, POD, and CAT) and proline accumulation. Furthermore, qPCR data suggested that *OsPEX11* acted as a positive regulator of salt tolerance by reinforcing the expression of several well-known rice transporters (*OsHKT2;1, OsHKT1;5, OsLti6a, OsLti6b, OsSOS1, OsNHX1*, and *OsAKT1*) involved in Na^+^/K^+^ homeostasis in transgenic plants under salinity. Ultrastructural observations of OsPEX11-RNAi seedlings showed that they were less sensitive to salt stress than WT and overexpression lines. These results provide experimental evidence that *OsPEX11* is an important gene implicated in Na^+^ and K^+^ regulation, and plays a critical role in salt stress tolerance by modulating the expression of cation transporters and antioxidant defense. Thus, OsPEX11 could be considered in transgenic breeding for improvement of salt stress tolerance in rice crop.

## Introduction

The gradual salinization of arable land is a serious constraint in the sustainable development of crop production. Among the cereals, rice is considered as one of the most salt sensitive crops (Zhu, 2001; Munns and Tester, 2008). With the development of biotechnology and genetic engineering, cloning and transferring of salt tolerant genes, could not only increase the utilization ratio of saline soils, but also supply a number of novel germplasm for breeding and sustainable food production (Geng et al., 2013; Dinneny, 2015). Thus, it is important to search and identify salt tolerant genes for successful development of high yielding cultivars for rice.

In the past decade, high-throughput transcriptomics and proteomics studies have provided immense data on gene function of plant salt tolerance (Zhuang et al., 2014). A few of important genes and/or proteins for osmolyte synthesis, ion channels and ROS scavenging enzymes have been founded from the previous studies (Parker et al., 2006; Jiang et al., 2007; Witzel et al., 2009; Kishor and Sreenivasulu, 2014), which have revealed the fundamental functions of the genes/proteins in crops’ response and adaptation to salinity. These bioinformatics data offer an integral view of salt-responsive genes in different plants. However, because of post-translational modifications and complication in salt response regulated networks, it is of utmost importance to elucidate exact biological function of these candidate genes/proteins under saline stress conditions.

Cyclophilins (CYPs), a class of highly conserved molecular chaperone, possessed peptidyl-prolyl *cis*-*trans* isomerase (PPIase) activity. These ubiquitous proteins are involved in a wide variety of biological processes such as protein assembly and transporting, RISC assembly and miRNA activity (Smith et al., 2009; Iki et al., 2012; Campos et al., 2013). In our previous report, we identified and characterized a rice cyclophilin (*OsCYP2*; Os02g0121300) that confers salt tolerance when overexpressed (Ruan et al., 2011). Until now, many cyclophilins genes have been discovered in *Glycine max, Arabidopsis thaliana*, and *O*. *sativa*, and they can be induced by a serial of abiotic stress, and heterologous expression confers toward multiple abiotic stresses (Mainali et al., 2014; Vasudevan et al., 2014; Lee et al., 2015).

Yeast two-hybrid is considered as a powerful tool to allow the identification of specific protein–protein interactions, which are more directly related to signal transduction processes under salt stress. However, the full list of peroxisomal proteins is not yet completely known (Dixit et al., 2010), which indicates the functions of peroxisome are still obscure. In this study, a peroxisomal biogenesis factor 11 family protein (OsPEX11; Os03g0302000) which interacted with OsCYP2 was screened by yeast two-hybrid assay in cDNA library of rice. Peroxisomal proteins are ubiquitous components of eukaryotic cells which are encoded by nuclear genes, synthesized on free cytosolic ribosomes and imported post-translationally (Gould and Valle, 2000; Nito et al., 2007; Kumar et al., 2014). They have been implicated in numerous metabolic processes, ranging from hydrogen peroxide metabolism to biosynthesis of lipids (Dixit et al., 2010; Pieuchot and Jedd, 2012; Odendall et al., 2014). Mutations of peroxisome biogenesis proteins in various eukaryotes result in serious developmental deficiencies and stress sensitivities (Aung and Hu, 2011; Burkhart et al., 2013; Cassin-Ross and Hu, 2014). They had been identified for involving in lipid catabolism, photorespiration and hormone biosynthesis in *Arabidopsis* (Nito et al., 2007). Our work is the first exploration on the biological functions of OsPEX11. The phenotype, physiological and expression level of candidate interacted protein (OsPEX11) were analyzed in overexpression and RNAi transgenic lines under salt stress. Therefore, these results will provide peroxisomal biogenesis factor mediated molecular and physiological responses of crop salt tolerance.

## Materials and Methods

### Construction of cDNA Library

Total RNA which was extracted from mix sample (leave, shoots, and roots) of 10-day-old wild type (WT) seedlings (*O*. *sativa* L. cv. Aichi-ashahi) was used to synthesize a cDNA library. The mRNA was purified by Dynabeads mRNA Purification Kit (Thermo Scientific, 61006). First and second strand synthesis and size fractionation were conducted according to the method of cDNA Library Construction Kit (Clontech, 634901) with minor modification. Then, cDNA library was directly cloned into the pGADT7AD vector encoding the GAL4 activation domain with *EcoR*I and *Xho*I. The size of inserted fragment was detected by using specific primers (Supplementary Table S1).

### Yeast Two-Hybrid Assay

Yeast two-hybrid analysis was performed in accordance with the Matchmaker Gold Yeast Two-Hybrid Kit (Clontech, 630489). The coding region of *OsCYP2* (519 bp) was amplified from rice leaves (*O. sativa* L. cv. Aichi-ashahi) by high-fidelity PCR, restricted and fused in-frame with GAL4 DNA binding domain into pGBKT7 for constructing bait vector. Then it was transformed into yeast strain Y2H through the lithium acetate method. After 3 days, auto-activation and toxicity assays were confirmed by SD/-Trp, SD/-Trp/X-α-gal, and SD/-Trp/X-α-gal/AbA selected plates. After that, the yeast two-hybrid screening between OsCYP2 and previous cDNA library was done according to the co-transformation protocol of Y2H strain. The candidate clones (blue) were selected by SD/-Trp/-Leu/X-α-gal/AbA plate. We patched out all the blue colonies that grew on SD/-Trp/-Leu/X-α-gal/AbA plate onto higher stringency SD/-Trp/-Leu/-His/-Ade/X-α-gal/AbA plate using yellow pipette tip. To increase the chance of rescuing the positive prey plasmid, we streaked 2–3 times for each selected single blue clone on SD/-Trp/-Leu/X-α-gal (no Aureobasidin A) plate. Then the candidate prey plasmid (blue clone) was rescued by using the Easy Yeast Plasmid Isolation Kit (Clontech, 630467) and sequenced with T7 primer. Co-transform BD or BD-OsCYP2 with rescued AD-prey plasmids into Y2H strains by small scale yeast transformation on selective media plates to distinguish positive interaction from false positive interaction.

### SDS-PAGE and GST Pull-down Assays

The genuine positive was further confirmed by GST pull-down assays. The *OsCYP2* and *OsPEX11* were cloned into pGEX-4T-1 and pET-28a vectors, respectively, for expressing fusion protein with glutathione-*S*-transferase (GST) and histidine (His), in *Escherichia coli* strain BL21. The MagneHis Protein Purification System (Promega, V8500) and MagneGST Pull Down System (Promega, V8870) were used for fused protein purification and GST pull-down, respectively. The purified GST, GST-OsCYP2 and His-OsPEX11 proteins were analyzed with 12% SDS-PAGE and stained by coomassie brilliant blue R-250. Western blotting signals were detected by Horseradish Peroxidase (HRP) DAB (3, 3-diaminobenzidine) staining with either the His tag antibody (Genescript, A00612) or GST tag antibody (Genescript, A00130).

### Plasmid Constructs and Plant Transformation

The full-length and partial cds of *OsPEX11* gene were cloned into pCAMBIA1300-Ubi and pCAMBIA1300-35S-RNAi vectors, respectively. The primers which contained different restriction enzyme sites were listed in Supplementary Table S1. Then, both of two vectors were transformed into *Agrobacterium tumefaciens* strain EHA105. Plant transformation was conducted into *O*. *sativa* L. cv. Aichi-ashahias previously described with minor modification (Hiei et al., 1994).

### Plant Growth and Quantitative RT-PCR

The seeds of WT, *OsPEX11* overexpression and RNAi were germinated and hydroponically grown in a greenhouse. The culture was maintained at 32°C/28°C, with the photoperiod of 16 h/8 h (light/dark). After 10 days, seedlings of each genotype were treated by 200 mM NaCl for 24 h and their phenotypes were identified. Six plants per treatment were sampled for the measurement of plant height, root length and leaves angle. Average values of these six plants were considered as one replicate. To examine the mRNA expression pattern of *OsPEX11* and seven crucial genes which code Na^+^ and K^+^ transport proteins, fresh leaves from each genotype were sampled for RNA isolation. Total RNA was extracted using Trizol (Thermo Scientific, 15596-026) according to the manufacturer’s instructions. Total RNA was used to synthesize the first strand cDNA with RevertAid^TM^ First-Strand cDNA Synthesis kit (Thermo Scientific, K1622). Quantitative real time PCR reactions were performed using three biological replicates for each different genotype (three technical replicates as one biological replicate) on Bio-Rad CFX 96. Primers used in this experiment were listed in Supplementary Table S1. The expression level of *OsPEX11* and other genes related to salt was calculated following Livak and Schmittgen (2001).

### Determination of Lipid Peroxidation and Antioxidant Enzyme

Lipid peroxidation was determined by malondialdehyde (MDA) contents according to the method of Hodges et al. (1999). Fresh leaves (0.5 g) of 10-day-old seedlings were homogenized in 10 ml of precooled potassium phosphate buffer (pH 7.0) by grinding with a mortar and pestle in an ice bath. The mixture was centrifuged at 4°C for 20 min at 12000 rpm. The supernatants were immediately used for the determination of various antioxidant enzymes. SOD, POD, and CAT activity were performed according to Dhindsa and Matowe (1981), Aebi (1984), and Rao et al. (1996), respectively. Moreover, proline content was measured according to Bates et al. (1973). For the determination of sodium and potassium ions, leaves were dried and ground. About 0.1 g of the ground leaf was digested with H_2_SO_4_ and H_2_O_2_, and then sodium and potassium contents were analyzed through atomic absorption spectrophotometry (Munns et al., 2010).

### Transmission Electron Microscopy

For electron-microscopic study, leaf fragments without veins (about 1 mm^2^) were fixed in 2.5% (v/v) glutaraldehyde in 0.1 M sodium phosphate buffer (PBS, pH 7.4) overnight and then washed three times with PBS. The samples were post fixed in 1% (m/v) OsO_4_ for 1 h and washed again three times with PBS. After that, the samples were dehydrated in a graded series of ethanol (50, 60, 70, 80, 90, 95, and 100%, v/v) for 15–20 min each and then in absolute acetone for 20 min. After dehydration, the samples were embedded in Spurr’s resin overnight. The specimens were heating at 70°C for 9 h, the ultra-thin sections (80 nm) were cut and mounted on copper grids for observation in the transmission electron microscope (TEM 1230EX, JEOL, Japan) at 60.0 kV.

### Statistical Analysis

The results presented here are the means of three replicates. Treatment means were compared by the analysis of variance (ANOVA) and using Tukey’s multiple range tests at the 5% level of significance.

## Results

### cDNA Library Quality and Yeast Two-Hybrid Screening

Total RNA and mRNA were extracted and purified from mix sample (shoot and root), respectively (Supplementary Figure 1). *EcoR*I and *Xho*I was introduced into the double strand cDNA which was synthesized by reverse transcription. Then, the synthesized cDNA was size fractionated to removing short fragments (Supplementary Figure 1). Then, double strand cDNA library was directional cloned into linearized pGADT7 AD vector with *EcoR*I and *Xho*I digested. The titer of cDNA library was more than 1.7 × 10^6^ cfu, and the size of inserted fragments were 1 kb approximately (Supplementary Figure 2).

**FIGURE 1:**
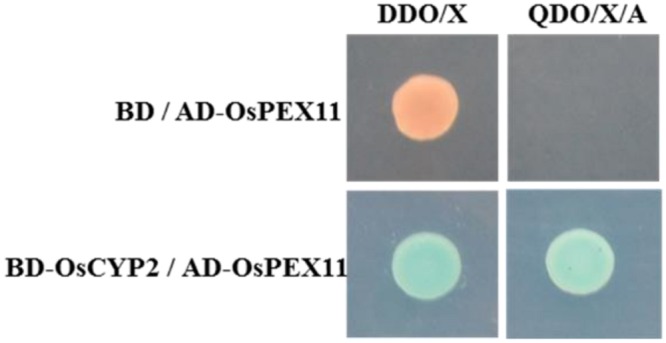
**OsCYP2 interacts with OsPEX11 protein by *in vivo* assay.** Yeast clones were grown on DDO/Xor QDO/X/A plates that contained X-α-gal. The blue color in the colony co-transformed with BD-OsCYP2 and AD-OsPEX11 indicates interaction between the two proteins. The empty pAD vector was used as negative control. DDO/X medium: SD/-Trp-Leu/X-α-gal; QDO/X/A medium: SD/-Trp-Leu-Ade-His/X-α-gal/AbA; AD-OsPEX11: pGADT7+OsPEX11; BD: pGBKT7 vector; BD-OsCYP2: pGBKT7+OsCYP2.

There was an identical size of Y2H strain on SD/-Trp plate containing bait vector (pGBKT7-*OsCYP2*) or not, meanwhile, pale blue and no clone on SD/-Trp/X-α-gal and SD/-Trp/X-α-gal/AbA plate, respectively (Supplementary Figure 3). That means the bait vector containing *OsCYP2* without auto-activation and toxicity, was suitable for yeast two-hybrid cDNA library screening. Then, 5 μg of bait and 10 μg of prey were used for yeast two-hybrid library co-transformation. The blue colonies that grew on DDO (SD/-Trp/-Leu/X-α-gal/AbA) plate were patched out onto higher stringency QDO (SD/-Trp/-Leu/-Ade/-His/X-α-gal/AbA) plate to eliminate the false interaction (Supplementary Figure 4). The result of sequencing and blast showed that rescued positive prey (No. 2) was a peroxisomal biogenesis factor 11 family protein (Os03g0302000). This prey protein was also identified by co-transforming into Y2H with pGBKT7 or pGBKT7-OsCYP2 on the selective media (**Figure 1**).

### OsCYP2 Directly Interacts with OsPEX11

**FIGURE 2:**
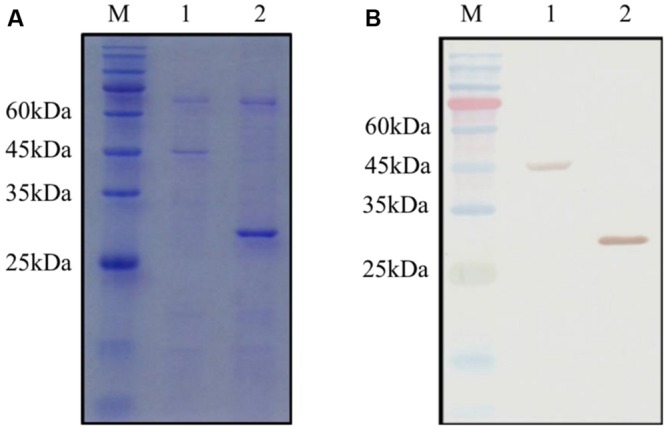
**Detection of GST (glutathione-*S*-transferase) and recombinant GST-OsCYP2.** The GST and GST-OsCYP2 proteins were extracted and purified from *E*. *coli* strain BL21 using MagneGST Pull Down System (Promega). The proteins were analyzed with 12% SDS-PAGE. **(A)** SDS-PAGE gel was stained by coomassie brilliant blue R-250. **(B)** Horseradish Peroxidase DAB staining was used in western blotting. 1: GST-OsCYP2. 2: GST. M: Pre-Stained Protein Marker.

**FIGURE 3:**
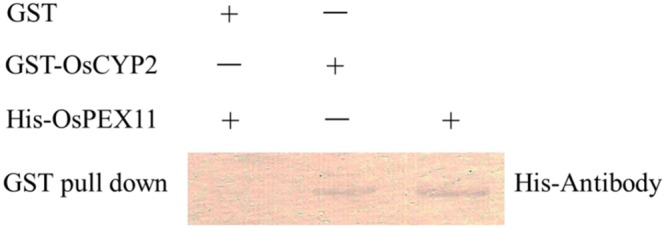
**Confirmation of interaction between OsCYP2 and OsPEX11 protein using *in vitro* assay.** Recombinant glutathione-*S*-transferase (GST) and GST-OsCYP2 was used as bait and incubated with 1 μg of prey protein (His-OsPEX11), respectively. After incubation, GST and GST-OsCYP2 were retrieved with glutathione beads, and the pulled-down proteins were detected on Western blots with antibodies to Histidine. GST: pGEX-4T-1; GST-OsCYP2: pGEX-4T-1+*OsCYP2*; His-PEX11: pET28a+*OsPEX11*.

The GST and GST-OsCYP2 protein were extracted from *E*. *coli* strain BL21 which contained pGEX4T-1 and pGEX4T-1 plus *OsCYP2*, respectively. Twenty-six kDa (GST) and 45 kDa (GST-OsCYP2) were showed by SDS-PAGE and coomassie brilliant blue staining (**Figure 2A**). We conducted the western blot to detect the specific signal by using HRP conjugated GST antibody and DAB staining (**Figure 2B**). In *in vitro* GST pull-down assay, OsCYP2 could directly interact with OsPEX11 (**Figure 3**).

### Phenotypic Characterization of OsPEX11 Transgenic Plants

**FIGURE 4:**
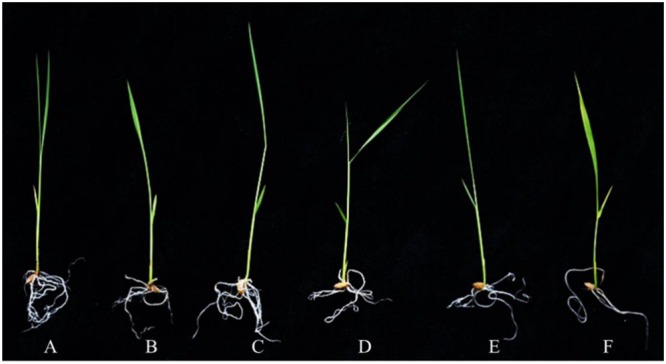
**The morphological changes of 10-day-old wild type (WT), *OsPEX11* over-expression (OE1) and RNAi (RNAi1) seedlings under control and salt stress (200 mM NaCl).** The seedlings were treated with 200 mM NaCl for 24 h. **(A)** WT, **(B)** WT + NaCl, **(C)** *35S*-*OsPEX11*, **(D)** *35S*-*OsPEX11* + NaCl, **(E)** RNAi-*OsPEX11*, and **(F)** RNAi-*OsPEX11* + NaCl.

**FIGURE 5:**
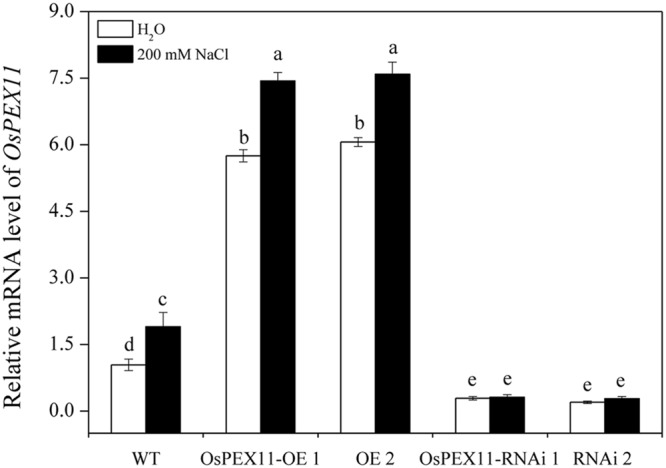
**Relative transcript level of *OsPEX11* responses to H_2_O and 200 mM NaCl stress in 10-day-old WT, overexpression (OE1 and OE2), and RNA interference (RNAi1 and RNAi2) lines.** The seedlings were treated with 200 mM NaCl for 24 h. Relative expression levels were measured by qRT-PCR analysis using *actin* as an internal standard. The relative fold expression of CK was considered as 1. Values are means of three biological replicates and significant differences between means, as determined by Turkey test (*P* < 0.05), are indicated by different letters.

To evaluate the stress response of transgenic plants, 10-day-old *OsPEX11* overexpression and RNAi seedlings were treated by 200 mM NaCl. After 24 h, leaves of WT and RNAi lines showed wilting, especially RNAi lines exhibited even more chlorosis, whereas the overexpressing plants remained normal growth condition (**Figure 4**). Moreover, the plant height was significantly decreased under 200 mM NaCl treatment in each genotype, however, root length and leave angles were significantly higher in *OsPEX11* overexpression seedlings compared with WT and *OsPEX11* RNAi (Supplementary Table S2). On the other hand, the transcript level of *OsPEX11* gene also showed significantly higher and lower expression in overexpression and RNAi lines, respectively, compared to that of WT. During salt stress treatment, *OsPEX11* was significantly induced in WT, and its expression level was up-regulated in overexpression lines (**Figure 5**).

### Sodium/Potassium Accumulation

**FIGURE 6:**
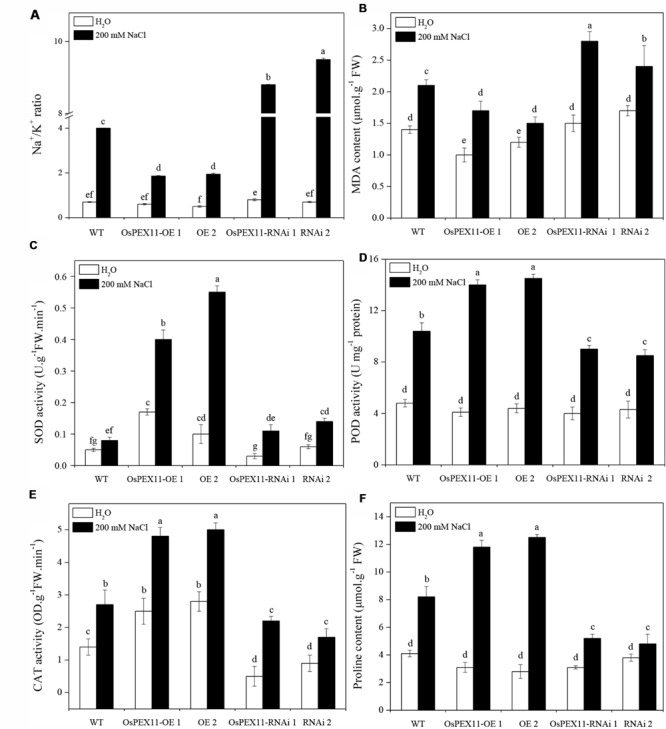
**Comparison of lipid peroxidation and ROS scavenging in leaves of 10-day-old WT and OsCYP2-transgenic (OE1 and OE2, RNAi1 and RNAi2) seedlings under control and salt stress.** The rice seedlings were treated with 200 mM of NaCl for 24 h. The Na^+^/K^+^ ratio **(A)** and activities of antioxidant enzymes MDA **(B)**, SOD **(C)**, POD **(D)**, CAT **(E)**, and proline content **(F)** were assayed. Values are means of three biological replicates followed by the same letter did not significantly differ at *P* ≤ 0.05 according to Turkey’s multiple range test.

Plant salt tolerance is mainly associated with the low maintenance of cytosolic Na^+^/K^+^ ratio. Thus, the sodium and potassium contents in the leaves of WT and transgenic plants were determined by atomic absorption spectrophotometry. There were no significant differences among three genotypes under control conditions, but in the presence of salt (200 mM NaCl), the Na^+^/K^+^ ratio was significantly elevated in *OsPEX11*-RNAi seedlings (**Figure 6A**). Compared with WT, the highest increase in Na^+^ concentration (2.5 fold) was observed in *OsPEX11*-RNAi plants. Similarly, K^+^ content maintained relatively higher in *OsPEX11*-OE seedlings (1.25 fold) (Supplementary Table S3). These results suggested that overexpression of *OsPEX11* can enhance salt tolerance and maintain a low Na^+^/K^+^ ratio through selective uptake of K^+^ over Na^+^.

### Physiological Responses to Saline Stress of Transformants

To further investigate the role of gene *OsPEX11* in plant tolerance to high salinity, we measured the effect of salt induced oxidative stress on MDA and ROS-scavenging anti-oxidative enzymes. As shown in **Figures 6B–F**, saline stress differentially modulated the accumulation of MDA and activities of SOD, POD, CAT enzymes and proline content in each type of seedlings. The MDA level was significantly lower in *OsPEX11* overexpression seedlings OE1 (15.35%) and OE2 (29.14%), while the SOD, POD, CAT enzyme activities were significantly increased (OE1: 5.00, 1.35, 1.78 fold; OE2: 6.88, 1.39, and 1.85 fold, respectively) as compared to WT plants. Accumulation of proline was accompanied by changes in ROS scavenging enzyme activities. Here, we found that the proline content was significantly elevated in *OsPEX11*-OE1 and OE2 seedlings (3.81 and 4.46 fold, respectively) under saline stress treatment. These results suggested that gene *OsPEX11* may have a better protection against salt induced ROS by dynamic modulation of antioxidant enzymes (SOD, POD, and CAT) and proline accumulation, which result in reduced lipid peroxidation under salt stress condition.

### Expression Pattern of Na^+^/K^+^ Transporter Proteins

**FIGURE 7:**
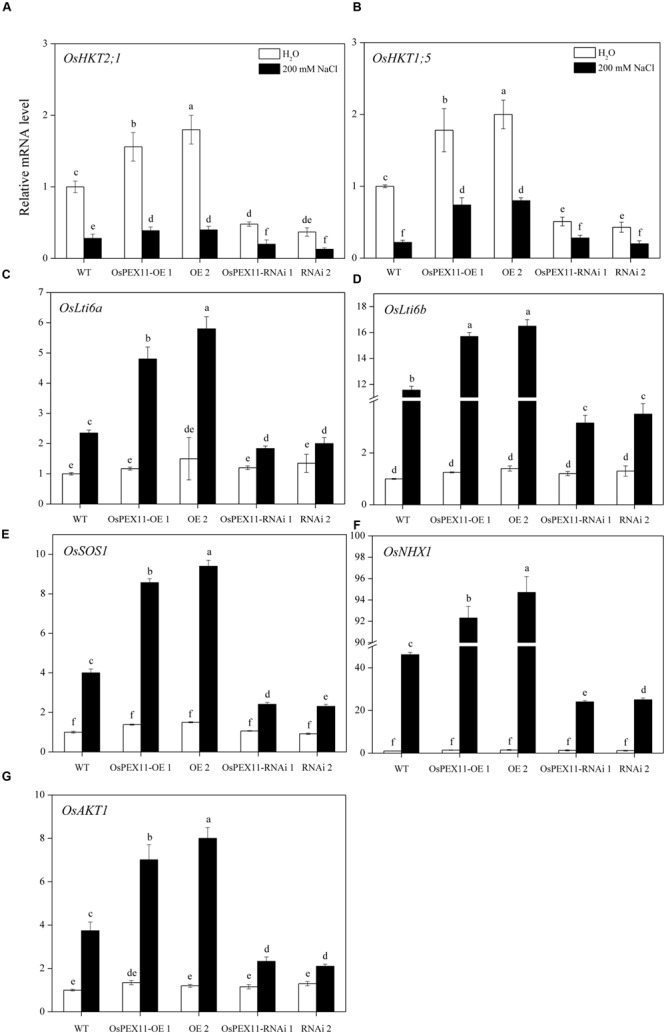
**Relative fold expression of the genes encoding Na^+^ and K^+^ transporter proteins. (A)** *OsHKT2;1*; **(B)** *OsHKT1;5*; **(C)** *OsLti6a*; **(D)** *OsLti6b*; **(E)** *OsSOS1*; **(F)** *OsNHX1*; **(G)** *OsAKT1* in the leaves of 10-day-old rice seedlings treated with 200 mM of NaCl. The relative fold expression of CK was considered as 1. An *actin* was used as internal standard. Values are the means of three biological replications ±SD. Variants possessing the same letter are not statistically significant at *P* < 0.05.

To better understand the mechanisms of underlying Na^+^/K^+^ accumulation variation in *OsPEX11* transgenic seedlings, we utilized qRT-PCR to detect the expression level of genes encoding Na^+^/K^+^ transport proteins. The data showed that except the other five genes, *OsHKT2;1* and *OsHKT1;5* (Na^+^ transporters) were significantly up/down-regulated in *OsPEX11* overexpression/RNAi lines under control condition. Saline stress treatment (200 mM NaCl) repressed the expression of the *OsHKT2;1* and *OsHKT1;5* (Na^+^ transporters) in each genotype (**Figures 7A,B**), however, *OsLti6a, OsLti6b* (two homologous *PMP3* genes involved in Na^+^ excess entry in plant cells), *OsSOS1* (Na^+^/H^+^ antiporter salt overly sensitive1), *OsNHX1* (vacuolar Na^+^, K^+^, and H^+^ antiporter) and *OsAKT1* (K^+^ transporter) performed an opposite effect (**Figures 7C–G**). In addition, *OsNHX1* was the most strongly induced (>20-fold) under salt stress overexpression seedlings compared with RNAi and WT (**Figure 7F**).

### Salinity Induces Ultrastructural Changes

**FIGURE 8:**
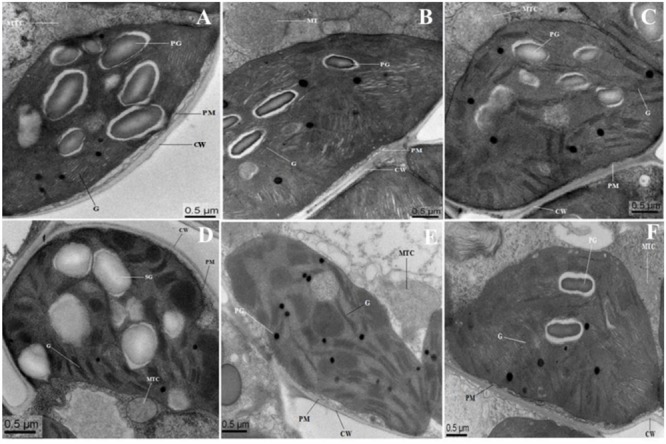
**Electron micrographs of leaf mesophyll cells of 10-day-old hydroponically grown seedlings of *O. sativa* under control condition and 200 mM NaCl treatment.** Under control condition, grana (G), mitochondria (MTC), and plastoglobuli (PG) well developed. TEM micrographs of leaf mesophyll cells of WT showed that ultrastructure of chloroplast is disrupted with enlarged size PG under 200 mM NaCl treatment **(D)**. Chloroplasts of OsPEX11-OE1 plants maintained normal shape under salt stress condition with dilated thylakoids arrangement **(E)**, whereas, chloroplasts of OsPEX11-RNAi1 plants were round in shape with fewer numbers of PG and swollen mitochondria (MTC) as compared to its respective control **(F)**. **(A–C)** control: WT (Left); OsPEX11-OE1 (Middle); OsPEX11-RNAi1 (Right); (D–F) 200 mM NaCl: WT (Left); OsPEX11-OE1 (Middle); OsPEX11-RNAi1 (Right).

**Figure 8** showed the representative TEM images of the chloroplast ultrastructure of WT and transgenic rice plants with/without saline stress conditions. In WT and *OsPEX11* overexpression plants, chloroplast was elliptical, the granum and stroma thylakoids were in an orderly arrangement, the lamellar structure was relatively tight, and the chloroplast envelope was intact with much larger size of starch grains under control condition (**Figures 8A,B**), while the structure of the chloroplast in *PEX11* RNAi plants was integrated, and the shape was round with swollen mitochondria (**Figure 8C**). Under saline stress condition, the shape of chloroplast in WT plants deformed with fewer thylakoid stacks of grana and lamella being observed. Additionally, the grana stacks were irregular and distributed unevenly with larger size of plastoglobuli, suggesting that the chloroplast structure was degraded (**Figure 8D**). The thylakoids of *OsPEX11*-OE1 plants were loose, the stroma thylakoids were in an orderly arrangement and chloroplast maintained relatively normal shape (**Figure 8E**). The chloroplast shape of *OsPEX11*-RNAi plants was round and dilated grana with fewer and abnormal shapes of mitochondria were also observed (**Figure 8F**).

## Discussion

Peroxisomes play key role in the regulation of metabolic process by modulating cellular redox homeostasis in a cell (Yun et al., 2012). It has been showed that peroxisomes can rapidly modify their metabolism and dynamics according to the subsequent ROS level in the cell. Mutations of peroxisome biogenesis proteins in various eukaryotes result in serious developmental deficiencies and stress sensitivities (Aung and Hu, 2011; Burkhart et al., 2013; Cassin-Ross and Hu, 2014). Under stress conditions, coordinated functioning of peroxisomal proteins provides highly dynamic responses of peroxisomal metabolism to adjust its redox metabolism at steady-state. In the present study, the overexpression of *OsPEX11* enhanced the growth and survival of transgenic plants under higher saline stress conditions, which further clarify it important role in reducing Na^+^ induced toxicity that protect the plants against saline stress.

Previous studies reported that enhanced activities of antioxidant enzymes (SOD, POD, and CAT) act as a coping strategy to scavenge ROS (Munns and Gilliham, 2015). Overexpression of genes involved ROS detoxification resulted in lower cellular damage, and the maintenance of photosynthetic energy capture under saline conditions (Roy et al., 2014). Similarly, *OsPEX11* gene substantially increased the antioxidant enzymes activities. It is likely that the peroxisome protein can suppress glycolate oxidase (Sousa et al., 2015) or harbor enzymes that can breakdown ROS (Antonenkov et al., 2010), and therefore led to decreased MDA contents as well as less damage to organelles membranes (Guan et al., 2015; Camejo et al., 2016). Proline is not only compatible osmolyte and osmoprotectant but also acts as a signaling molecule to modulate ion balance, protect enzyme activity and trigger the expression of specific genes, which are essential for plant recovery from stresses (Liu et al., 2015). In the present study, we found enhanced accumulation of proline in overexpression plants compared to RNAi plants (**Figure 6**). The increased content of proline may help to decrease Na^+^ toxicity in overexpression plant as compared to the RNAi plants (Islam et al., 2015, 2016). Therefore, *OsPEX11* overexpression could alleviate ion toxicity and oxidant damage in transgenic plants by enhancing proline accumulation and antioxidant defense.

The accumulation of K^+^ and Na^+^ in plants is important parameter to understand salt tolerance mechanisms (Noreen et al., 2010). In the present investigation, overexpression plants accumulated high K^+^ and maintained lower Na^+^/K^+^ ratio and Na^+^ accumulation under stress conditions as compared to RNAi plants. The higher accumulation of Na^+^ in RNAi plant might be due to enhanced accumulation of Na^+^ in roots, passive diffusion of sodium ions from damaged membranes and reduced efficient mechanism of sodium diffusion (Islam et al., 2015). Thus, *OsPEX11* overexpression could improve the growth of rice seedlings by reducing Na^+^ uptake under saline stress condition as compared to the RNAi plants. Furthermore, the ability of the plants to restrict the transportation and accumulation of Na^+^ is considered as a critical aspect of plant salt tolerance/adaptation (Apel and Hirt, 2004; Foyer and Noctor, 2005; Munns and Tester, 2008). To understand the salt-tolerant mechanism in *OsPEX11* overexpression plants, we compared the transcript levels of marker genes related to Na^+^ and K^+^ regulation under control and stress conditions (**Figure 7**). The *OsPEX11* overexpression plants exhibit lower leaf Na^+^ concentration showed better adaptation to the salinity. This controlled entry of Na^+^ to the leaf is often associated with the lowered Na^+^/K^+^ ratio (**Figure 6A**), which is vital for the normal functioning of a cell under saline stress conditions (Su et al., 2015). Na^+^ exclusion/restriction is usually under the control of different Na^+^ transport proteins. Such as, high affinity potassium transporter (*HKT*) genes encode Na^+^ selective transporter proteins which demonstrated essential salinity tolerance mechanism in different crop plants including rice (Hamamoto et al., 2015). To understand underlying mechanism of restricted Na^+^ transport in the leaves of transgenic and WT plants, we analyzed the expression of *OsHKT1;5* both under control and salt stress conditions. *OsPEX11* overexpression plants showed considerable induction in transcript level of *OsHKT1;5* as compared to the RNAi plants, which may suggest that downregulation of *OsHKT1;5* expression in RNAi plants may be the reason of higher accumulation of Na^+^ in leaves as compared to the overexpressed plants, that restricted the unregulated Na^+^ transport to the leaves and protect the plants from growth impairment due to Na^+^ toxicity (**Figure 7**). Previously, decreased expression of *HKT1;5* through RNAi in bread wheat was linked with increased Na^+^ accumulation in the leaves, which suggest a critical role of *HKT1;5* transporter in the restriction of Na^+^ transport from root to leaves in saline stress plants (Byrt et al., 2014).

Plasma membranes proteins 3 (PMP3) are conserved hydrophobic proteins induced in wide range of abiotic stress conditions, suggesting their significant role in membranes stability under stress conditions (Chidambaram et al., 2015). The overexpression of PMP3 homolog proteins in *Arabidopsis* and *Avicennia marina* plants show lowered shoot Na^+^ levels and enhanced plant performance under stress conditions (Mitsuya et al., 2006; Chidambaram et al., 2015). In the present study, the expression level of two rice homologous *PMP3* genes, *OsLti6a* and *OsLti6b* was downregulated under saline stress condition in RNAi plants as compared to the overexpression and WT plants, which clearly indicate the involvement of these genes in Na^+^ entry in the RNAi plants (**Figure 7**). Such as, a loss of function of PMP3 in yeast mutants resulted in Na^+^ accumulation, salt sensitivity and membrane hyperpolarization (Nylander et al., 2001). Additionally, Mekawy et al. (2015) suggested that reduction in the expression levels of PMP3 genes may facilitate Na^+^ entry in sensitive rice cultivar as compared to the resistant one. The enhanced activation of PMP3 proteins in *OsPEX11* overexpression plants may be due to the induction of other Na^+^ transporter proteins, because PMP3 proteins contribute indirectly to cation homeostasis within cells by interaction with other ion transporters (Fu et al., 2012). Plasma membrane Na^+^/H^+^ exchanger (*SOS 1*) and tonoplast Na^+^/H^+^ exchanger (*NHX*) are considered as main transporters mediating the eﬄux and compartmentalization of Na^+^ under saline stress conditions (de la Garma et al., 2015).

Both antiporters are ubiquitous membrane proteins that catalyze the electroneutral exchange of Na^+^ or for H^+^ across the membrane, thereby playing important roles in cellular Na^+^/K^+^ homeostasis. *SOS1* is not only involved in Na^+^ exclusion from cytoplasm but also maintain optimum level of Na^+^ by xylem loading under low salt stress, while Na^+^ removal from xylem under saline stress conditions. In the present investigation, upregulation of *OsSOS1* in overexpression plants as compared to the RNAi and WT plants probably facilitated exclusion of toxic Na^+^ into root apoplast and thus resulted in higher K^+^/Na^+^ ratio of leaves (**Figure 6A**). Additionally, Na^+^/ K^+^/H^+^ antiporter *NHX1* is involved in the intracellular Na^+^/K^+^ sequestration in vacuoles, depending on the salt concentration in the cell. We observed that *NHX1* gene expression levels were more upregulated in overexpression plants which may be the reason of higher accumulation of K^+^ uptake into the vacuole and lower Na^+^ concentration in cytoplasm, which relieves the toxic effect on cytosolic enzymes, maintaining turgor pressure and cell expansion under saline stress conditions (Cao et al., 2016).

Moreover, ultrastructural observations of leaf mesophyll cells were also conducted to confirm the salt tolerance of *OsPEX11* gene. Because, chloroplast and mitochondria are sensitive to the salinity. Salinity may change the functionality and integrity of chloroplast, that can affect energy metabolism of the mesophyll cells. The ultrastructural alternations in the chloroplast and mitochondrial structures in our study are consistent with the previous reports (Tripathy et al., 2015), suggesting that the accumulation of excess Na^+^ in RNAi and WT plants damaged the chloroplasts ultimately causing decreased photosynthesis efficiency at the whole plant level and thereby reduced growth and biomass production. Because, productivity and maintenance of the structural integrity of chloroplast is directly linked with the conversion of light energy during photosynthesis. Taken together, the overexpression of *OsPEX11* gene enhanced the growth and survival of plants under salinity but further research is needed to explore the relationship between *OsPEX11* and salt stress tolerance using advanced physiological and molecular technologies.

## Conclusion

In our present study, a novel prey protein (OsPEX11) was screened and identified using *in vivo* (yeast two-hybrid) and *in vitro* (GST pull-down) assays. Under saline stress, leaves of *OsPEX11-RNAi* lines showed wilting and exhibited even more chlorosis compared to *OsPEX11* overexpressing plants. *OsPEX11* gene may have a better protection against salt induced ROS by dynamic modulation of antioxidant enzymes (SOD, POD, and CAT) and proline accumulation, which can reduce lipid peroxidation under salt stress condition. We also demonstrated that *OsPEX11* overexpression can better protect plants from saline stress by restricting the entry of excess Na^+^ through dynamic regulation of Na^+^/K^+^ transporter proteins and its subsequent sequestration through *NHX1* in vacuoles. Moreover, ultrastructural observations of *OsPEX11* overexpression seedlings demonstrated that they had better tolerance to salt stress than RNAi and WT plants.

## Author Contributions

Conceived and designed the experiments: HL and WZ. Performed the experiments: PC, FI, LL, and MF. Analyzed the data: HL, WZ, LL, SR, and PC. Contributed reagents/materials/analysis tools: SR, WZ, HL, and MF. Wrote and revised the paper: PC, HL, FI, and WZ.

## Conflict of Interest Statement

The authors declare that the research was conducted in the absence of any commercial or financial relationships that could be construed as a potential conflict of interest.
