# Supplementary material for: OsPEX11, a Peroxisomal Biogenesis Factor 11, Contributes to Salt Stress Tolerance in Oryza sativa
Source: Front Plant Sci. 2016 Sep 15;7:1357. doi: 10.3389/fpls.2016.01357 (PMC5024708; doi:10.3389/fpls.2016.01357)
Supplement: Supplementary file 1 [file Table_1.DOC]

**Supplementary Materials**

Table S1. Primers used in this study.

| Primer | 5’ 3’ |
| --- | --- |
| Inserted fragment-F | GGAGTACCCATACGACGTACC |
| Inserted fragment-R | TATCTACGATTCATCTGCAGC |
| BD-OsCYP2-F | CGGGAATTCTCTGTGAAATTCGCAAAACCC |
| BD-OsCYP2-R | ACGCGTCGACACACCACCACCACCTCCTCCT |
| GST-OsCYP2-F | CGGAATTCATGTCGAACACGAGGGTGTT |
| GST-OsCYP2-R | CCGCTCGAGCTAGGAGAGCTGGCCGCAGT |
| His-OsPEX11-F | CGCGGATCCATGGCCGCCGCCGCCGCCGC |
| His-OsPEX11-R | CCGCTCGAGGCACGAATTCCAATTCTTGT |
| OsPEX11-OE-F | CGGGGTACCCTCTCTTCCACGCAGCAACC |
| OsPEX11-OE-R | AACTGCAGTCAGCACGAATTCCAATTCT |
| OsPEX11-RNAi-F1 | CGCGGATCCGCGTCTACTACTTCCTCG |
| OsPEX11-RNAi-R1 | GGACTAGTGACTCCAGTTTGCCGATC |
| OsPEX11-RNAi-F2 | CGAGCTCGCGTCTACTACTTCCTCG |
| OsPEX11-RNAi-R2 | CGGGGTACCGACTCCAGTTTGCCGATC |
| Actin-F | GACCTTGCTGGGCGTGAT |
| Actin-R | GTCATAGTCCAGGGCGATGT |
| qRT-OsPEX11-F | GCGTCTACTACTTCCTCG |
| qRT-OsPEX11-R | GACTCCAGTTTGCCGATC |
| OsHKT2;1-F | TGCATTCATCACTGAGAGGAG |
| OsHKT2;1-R | GGTGCAGTTTCTGCAACCTC |
| OsHKT1;5-F | CCCATCAACTACAGCGTCCT |
| OsHKT1;5-R | AGCTGTACCCCGTGCTGA |
| OsLti6a-F | CCTTCCAAGGTGATGGTGAA |
| OsLti6a-R | CCGTCCAAAGAACCAGAAAA |
| OsLti6b-F | GCTCCAAACCGCTTCATCTA |
| OsLti6b-R | CAAGAATTGGAGCACTCAGGA |
| OsSOS1-F | ATACTGAGTGGGGTTGTTATTGC |
| OsSOS1-R | AAAGGTAAATTTCAAAAGGTACATGG |
| OsNHX1-F | AATGATCACCAGCACCATCA |
| OsNHX1-R | AAGGCTCAGAGGTGACAGGA |
| OsAKT1-F | GAAACGAGCAATGCGTCAG |
| OsAKT1-R | CTTCTCACACAGCGCTTCC |

Table S2. Effect of 200 mM NaCl treatment on morphological parameters in 10-day-old wide type and *OsPEX11* transgenic plants

| Genotype | Height (cm) | | Primary root length (cm) | | Leave angle (°) | |
| --- | --- | --- | --- | --- | --- | --- |
|  | H_2_O | NaCl | H_2_O | NaCl | H_2_O | NaCl |
| WT | 7.50b | 6.50d | 5.10b | 4.90b | 20c | 20c |
| OsPEX11-OE 1 | 8.80a | 7.00c | 5.40a | 5.60a | 25b | 33a |
| OsPEX11-OE 2 | 9.00a | 7.10c | 5.45a | 5.73a | 23b | 30a |
| OsPEX11-RNAi 1 | 7.40b | 5.80e | 4.95b | 5.00b | 25b | 26b |
| OsPEX11-RNAi 2 | 7.40b | 5.90e | 4.98b | 4.87b | 23b | 24b |

Table S3. Concentration of Na^+^ and K^+^in leaves of wide type and *OsPEX11* transgenic plants under control (H_2_O) andsalinity stress (200MmNaCl)

| Genotype | Na^+^ concentration  (μM g/DW) | | K^+^ concentration  (μM g/DW) | |
| --- | --- | --- | --- | --- |
|  | H_2_O | 200 mMNaCl | H_2_O | 200 mMNaCl |
| WT | 1.40 d | 5.80 b | 2.00 b | 1.45 c |
| OsPEX11-OE 1 | 1.20 d | 4.10 c | 2.00 b | 2.20 b |
| OsPEX11-OE 2 | 1.40 d | 3.88 c | 2.80 a | 2.00 b |
| OsPEX11-RNAi 1 | 1.50 d | 13.20 a | 1.88 b | 1.50 c |
| OsPEX11-RNAi 2 | 1.45 d | 14.73 a | 2.10 b | 1.55 c |

The same letters within a column indicate there was no significant difference at a 95% probability level.


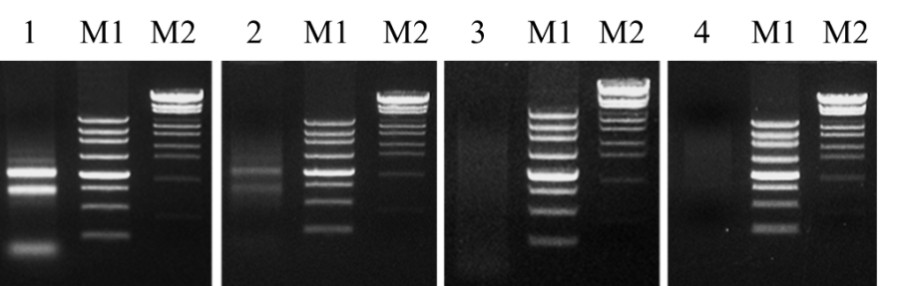


Supplementary Fig. 1 Construction of cDNA library in *Oryza sativa* L. cv. Aichi-ashahi.

1: Total RNA, 2: purified mRNA, 3: double strand cDNA, 4: size-fractionated cDNA

M1:250bp DNA Ladder marker, M2: *λ*-*Eco*T14 I digest.


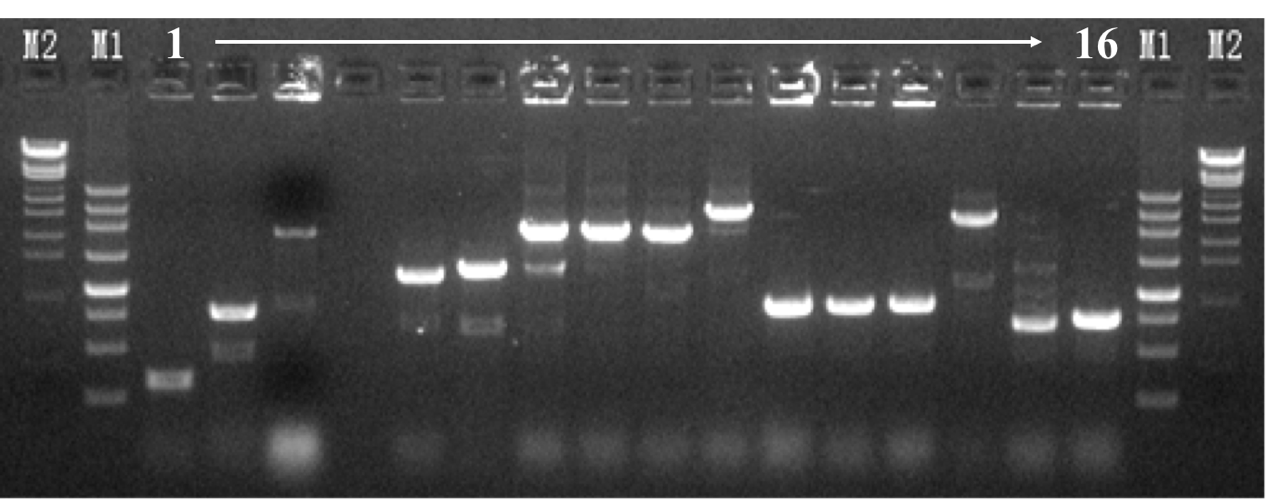


Supplementary Fig. 2 Identification of insert in rice cDNA library.

1-16: insert fragments, M1:250bp DNA Ladder marker, M2: *λ*-*Eco*T14 I digest.


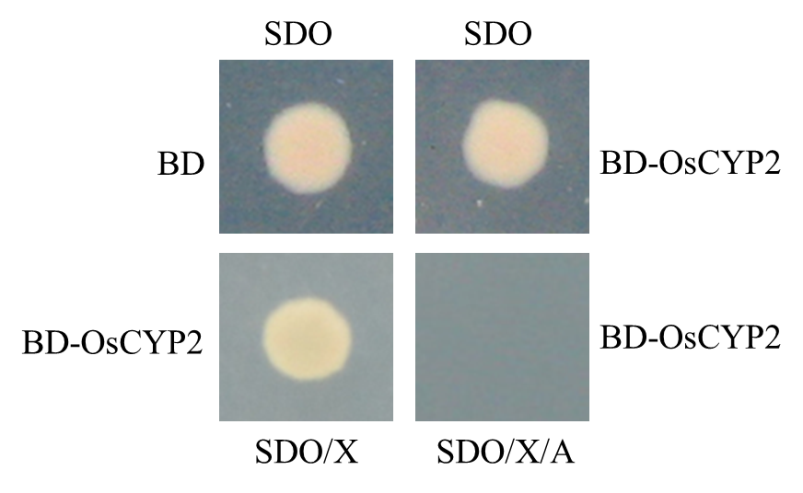


Supplementary Fig. 3 Autoactivation and toxicity testing for *OsCYP2*.

SDO: SD/-Trp, SDO/X: SD/-Trp/X-α-gal, SDO/X/A: SD/-Trp/X-α-gal/AbA

BD: pGBKT7, BD-OsCYP2: pGBKT7+*OsCYP2*.


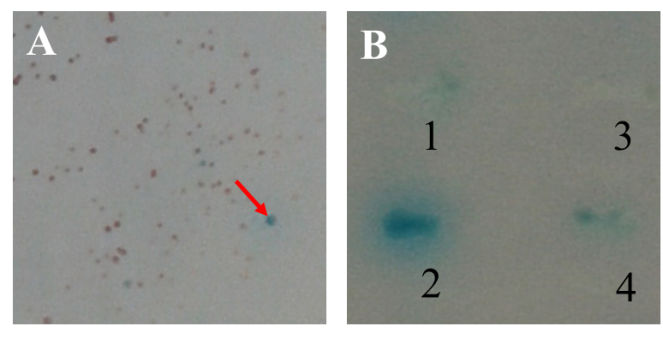


Supplementary Fig. 4 Screening of interacted proteins in rice cDNA library by yeast two-hybrid.

A: Mating diploids on SD/-Trp-Leu/X-α-gal/AbA; the red arrow represent positive interaction

B: High stringency screening on SD/-Trp-Leu-Ade-His/X-α-gal/AbA; 1-4: independent interaction
